# Do Students’ Motivation Influence Interprofessional Competencies? The Mediating Role of Team Efficacy

**DOI:** 10.1007/s40670-026-02789-2

**Published:** 2026-06-23

**Authors:** John Ian Wilzon Dizon, Yue Lei, Eileen Tipoe, Miu Yung Olivia Ngan, Charlotte Tsang, Pauline Yeung Ng, Dana Vackova, Linda Chan, Arkers Kwan Ching Wong, Lily Yuen Wah Ho, Zoe Ng, Jody Kwok Pui Chu, Franco Wing Tak Cheng, Pauline Yuen Ping Wan, Kevin K. Tsia, Benney Yiu Cheong Wong, Chad Wing Nga Chan, Doris Yin Kei Chong, Grace Szeto, Pauline Luk, Chor Yin Lam, Rachel Lai Chu Kwan, Fraide A. Ganotice

**Affiliations:** 1 Li Ka Shing Faculty of Medicine, Bau Institute of Medical and Health Sciences EducationThe University of Hong Kong, Hong Kong SAR, China; 2 School of Economics and Finance, Queen Mary University of London, London, England, UK; 3 Medical Ethics and Humanities Unit, School of Clinical Medicine, Li Ka Shing Faculty of Medicine, The University of Hong Kong, Hong Kong SAR, China; 4 Department of Rehabilitation Sciences, Faculty of Health and Social Sciences, The Hong Kong Polytechnic University, Hong Kong SAR, China; 5 Critical Care Medicine Unit, School of Clinical Medicine, Li Ka Shing Faculty of Medicine, The University of Hong Kong, Hong Kong SAR, China; 6 School of Public Health, Li Ka Shing Faculty of Medicine, The University of Hong Kong, Hong Kong SAR, China; 7 School of Nursing, Faculty of Health and Social Sciences, The Hong Kong Polytechnic University, Hong Kong SAR, China; 8 School of Nursing, Li Ka Shing Faculty of Medicine, The University of Hong Kong, Hong Kong SAR, China; 9 Department of Pharmacology and Pharmacy, Li Ka Shing Faculty of Medicine, The University of Hong Kong, Hong Kong SAR, China; 10 Department of Social Work & Social Administration, Faculty of Social Sciences, The University of Hong Kong, Hong Kong SAR, China; 11 Department of Electrical and Electronic Engineering, Faculty of Engineering, The University of Hong Kong, Hong Kong SAR, China; 12 Department of Physiotherapy, School of Nursing and Health Sciences, Hong Kong Metropolitan University, Hong Kong SAR, China; 13 School of Medical and Health Sciences, Tung Wah College, Hong Kong SAR, China; 14 Department of Orthopaedic & Traumatology, School of Clinical Medicine, Li Ka Shing Faculty of Medicine, The University of Hong Kong, Hong Kong SAR, China

**Keywords:** Interprofessional education, Academic motivation, Competence development, Team efficacy, Mediation analysis

## Abstract

**Background:**

While academic motivation drives individual learning, its role in developing collaborative competencies in interprofessional education (IPE) remains underexplored—a critical gap given healthcare’s increasing reliance on interprofessional teams.

**Objective:**

Grounded in self-determination theory, this study examined how academic motivation relates to interprofessional competencies (IPEC) development and whether team efficacy (TE) mediates this relationship.

**Method:**

Data were collected from a cohort of undergraduate students from three disciplines from two universities in Hong Kong at the end of their month-long participation on either of the two IPE modules (Fracture and Emerging Respiratory Infection). Participants’ academic motivation (knowledge and accomplishment), perception of their team’s efficacy, and competencies (values and ethics, roles and responsibilities, communication, teamwork) were measured using standardized self-report scales.

**Results:**

Mediation analyses via PROCESS Macro Model 4 revealed distinct patterns for the two motivation dimensions. Both academic knowledge (AMK) and accomplishment motivation (AMA) directly predicted all four IPEC competency domains (b = 0.35–0.45, *p* < 0.001) and team efficacy (b = 0.15–0.20, *p* < 0.001–0.05). Team efficacy predicted all IPEC domains (b = 0.15–0.20, *p* < 0.05) and mediated the AMA-IPEC relationship across all four domains (indirect effects b = 0.06–0.07, 95% CIs excluding zero). However, perceived team efficacy mediated the AMK-IPEC relationship only for Roles and Responsibilities (b = 0.07, 95% CI [0.01, 0.16]). Accomplishment-motivated students thus develop interprofessional competencies through both team-mediated and individual pathways, while knowledge-motivated students rely primarily on direct learning.

**Conclusion:**

Given the importance of team efficacy and motivation in developing interprofessional competencies, IPE curricula must nurture both motivation types while recognizing their distinct pathways: structuring team tasks to leverage accomplishment motivation’s natural team engagement, while providing individual learning opportunities for knowledge-motivated students to minimize role ambiguity.

**Supplementary Information:**

The online version contains supplementary material available at https://doi.org/10.1007/s40670-026-02789-2.

## Introduction

Communication and teamwork failures remain a leading contributor to preventable patient harm worldwide. The World Health Organization’s *Global Patient Safety Report 2024* [1] estimates that more than one in ten patients experience harm during healthcare delivery, and at least half of these adverse events are preventable. The Joint Commission [2] documented 1,575 sentinel events (defined as preventable patient safety events resulting in death or severe harm) in the United States, with communication breakdowns identified among the leading contributing factors. Hong Kong’s Hospital Authority [3] reports a similar pattern where communication deficits are primary contributors to sentinel events. In primary care globally, an estimated 7% of patients experience harm, with communication and coordination failures featuring prominently in the underlying causes [1]. These persistent failures have driven healthcare education to embrace interprofessional education (IPE) as a pedagogical strategy for developing the collaborative competencies needed to prevent them [4].

IPE brings students from different health disciplines together during their pre-licensure years to learn with, from, and about each other [4]. Its core aim is the development of four interprofessional competencies (IPEC) [5]: values and ethics for interprofessional practice (VE), roles and responsibilities (RR), interprofessional communication (IC), and teams and teamwork (TT). Despite four decades of accumulated practice, how students actually acquire these competencies during IPE remains theoretically underspecified. The present study addresses one part of this gap: how academic motivation, an established driver of individual learning, fosters collaborative competencies that emerge through team interaction. We test whether students’ perceived team efficacy serves as a psychological mechanism that connects individual motivation to IPEC competency development.

### Academic Motivation in Educational and IPE Contexts

Within self-determination theory (SDT) [6, 7], academic motivation reflects the extent to which learners engage in academic activities with a sense of volition and value. A recent meta-analysis of 344 samples involving over 223,000 students reported that autonomous forms of motivation (intrinsic motivation and identified regulation) account for substantial variance in academic achievement, behavioural engagement, and persistence across diverse educational contexts [6]. Intrinsic motivation, the most self-determined form, is theoretically partitioned into subtypes [8]. Intrinsic motivation to know (AMK) is characterised by the pleasure of learning and discovery. Intrinsic motivation toward accomplishment (AMA) is characterised by satisfaction in mastery and surpassing one’s own limits. Both subtypes reflect autonomous, self-determined motivation and are typically positively correlated in academic samples.

In IPE specifically, an emerging body of research demonstrates that autonomous motivation predicts important learning and collaboration outcomes. A study found that students’ sense of autonomy positively predicted behavioural engagement, team effectiveness, collective dedication, and IPE goal achievement among undergraduate healthcare students in Hong Kong [9]. Another study reported sustained gains in medical and nursing students in the Netherlands [10]. These findings establish that motivation matters in IPE, but they leave open a more specific question: how does motivation translate into the four IPEC competency domains, which by definition emerge from interaction with teammates from other professions rather than from individual study? SDT articulates how need satisfaction drives individual achievement, but the theory has been developed and tested predominantly in individual learning contexts. How autonomous motivation operates within the interdependent, socially embedded processes of interprofessional teamwork remains theoretically underdeveloped.

### Team Efficacy in Collaborative Learning Contexts

Team efficacy, defined as the belief in a team’s collective capability to organise and execute the courses of action required to produce given levels of attainment [11, 12], is a robustly supported predictor of group functioning. Meta-analytic evidence establishes both the magnitude and the boundary conditions of this relationship. For example, a meta-analysis synthesizing 96 studies and 6,128 groups across organisational and educational settings reported a corrected mean correlation of ρ = 0.35 between collective efficacy and group performance [13]. In addition, another meta-analysis pooling 67 studies and 256 effect sizes found team-level effects of ρ = 0.41 and demonstrated that task interdependence moderates the team efficacy–performance relationship: when team tasks require coordinated effort, perceived team efficacy is a stronger predictor of performance [14]. IPE contexts exemplify such task interdependence by design, since interprofessional case management cannot be accomplished by any single discipline working in isolation.

In IPE settings specifically, related team-level constructs have been linked to collaborative outcomes. Collective efficacy was found to fully mediate the relationship between team cohesiveness and three collaboration outcomes (teamwork satisfaction, satisfaction with the team experience, and IPE goal attainment) among healthcare students in Hong Kong [15], identifying collective efficacy as the psychological mechanism through which cohesive teams achieve better collaborative outcomes. Another study observed positive associations between competencies and team efficacy in an interprofessional training ward for medical and nursing students in Germany [16]. These studies establish team efficacy as a meaningful construct in IPE but have used correlational designs that cannot test team efficacy as a mechanism linking individual psychological characteristics to competency development.’’.

### Theoretical Framework

The present study draws together two complementary theoretical traditions to articulate how individual motivation may translate into collaborative competencies through perceptions of team functioning.

The first is self-determination theory (SDT) [7, 17]. SDT proposes that human motivation is sustained by the satisfaction of three basic psychological needs: autonomy (a sense of initiative and ownership in one’s actions), competence (a sense of mastery and effective engagement with one’s environment), and relatedness (a sense of meaningful connection with others). When these needs are supported in a given context, learners exhibit autonomous forms of motivation, which in turn drive engagement, persistence, and skill acquisition. SDT further proposes, through Vallerand’s tripartite hierarchical model [18, 19], that intrinsic motivation can be partitioned into subtypes oriented around different sources of satisfaction. AMK is anchored primarily in the competence need, with autonomy entering through self-directed choice of learning content. AMA is also anchored in competence but extends, in collaborative settings, to the relatedness need, since accomplishment in interprofessional tasks is necessarily achieved through coordinated team performance. This theoretical distinction grounds our examination of AMK and AMA as separable subtypes of intrinsic motivation, while acknowledging that they are not predicted by SDT to operate identically across team-mediated and individual learning pathways.

The second is Bandura’s social cognitive theory of efficacy beliefs [11, 20, 21], which extends self-efficacy from the individual to the collective level. Bandura proposes that just as individuals form beliefs about their personal capabilities, members of interdependent groups form beliefs about their team’s collective capability to achieve shared goals. These efficacy beliefs are theorised to shape behavioural choices, effort allocation, persistence in the face of difficulty, and recovery from setbacks, with team-level effects most pronounced under conditions of task interdependence (see Sect. Team Efficacy in Collaborative Learning Contexts).

We integrate these two frameworks by proposing that academic motivation, an individual-level psychological construct grounded in SDT, may influence the development of interprofessional competencies through students’ perceived team efficacy, an individual-level perception of the team’s collective capability grounded in social cognitive theory. SDT explains why motivated learners would engage proactively in team processes; social cognitive theory explains how perceptions of team capability shape the willingness to engage in the specific behaviours through which IPEC competencies are practised and refined. The satisfaction of relatedness in SDT, which arises from meaningful connection with others, is partly indexed by students’ perceptions of their team’s functioning. Within this integrated framework, students’ perceived team efficacy serves as a psychological bridge that connects individual motivation to collaborative competency development.

### The Present Study

The present study tests whether students’ perceived team efficacy mediates the relationship between academic motivation and IPEC competency development. Three considerations guide this work. First, although prior research has examined motivation in IPE [9, 10] and team efficacy in IPE [15, 16, 22] separately, whether these two constructs operate through a shared psychological pathway has not been empirically tested. By specifying students’ perceived team efficacy as a mediator between motivation and IPEC, we test a theoretically integrated model rather than treating motivation and team dynamics as independent predictors of competency development. Second, although AMK and AMA are theoretically distinguishable subtypes of intrinsic motivation [18, 19], how each operates in IPE settings has not been systematically compared. We treat the AMK–AMA comparison as an open empirical question and impose no directional prediction about which subtype should channel more strongly through students’ perceived team efficacy. Third, the design is cross-sectional, with academic motivation, students’ perceived team efficacy, and self-rated competencies measured at the end of a four-week IPE module. The mediation analyses therefore test a theoretically specified indirect effect at a single time point and do not establish temporal precedence among the constructs. We therefore interpret our findings based on these considerations.

**Fig. 1:**
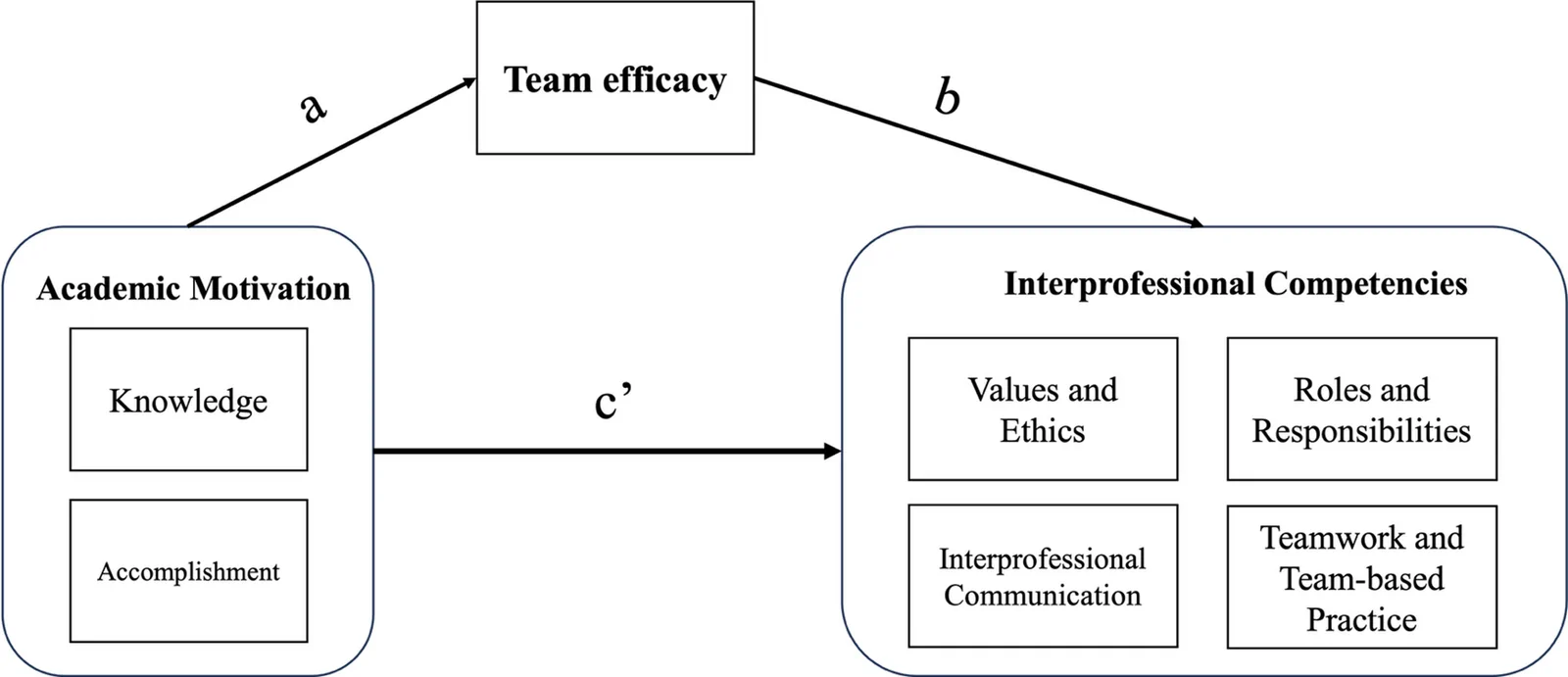
The hypothesized conceptual framework illustrates academic motivation orientations predicting the four interprofessional education competencies, mediated by team efficacy. *Note*: All paths are hypothesized to be positive

The proposed mediation is hypothesised to be partial rather than complete. Motivated learners engage in both team-based learning (which is captured by the team-mediated pathway through students’ perceived team efficacy) and individual learning pathways including independent study of interprofessional concepts, observation of role models in interprofessional teams, and reflection on professional identity [7]. Both pathways may contribute to IPEC development, and we test their relative contributions through a direct effect (individual pathway) and an indirect effect through students’ perceived team efficacy (team-mediated pathway). The hypothesised model is depicted in Fig. 1.

### Research Questions and Hypotheses

Specifically, we proposed the following research questions (RQ) and hypotheses (H):

- RQ1: Does students’ academic motivation predict their interprofessional competencies?
- H_1_: Academic motivation positively predicts interprofessional competencies. Grounded in self-determination theory, learners with higher intrinsic motivation exhibit greater engagement in skill acquisition [7], including interprofessional collaboration [23].
- RQ2: Does students’ academic motivation predict their perceived team efficacy?
- H_2_: Academic motivation (both knowledge and accomplishment orientations) positively predicts students’ perceived team efficacy. Motivated learners are more likely to contribute proactively to team tasks, fostering cohesion and shared goals [12].
- RQ3: Does students’ perceived team efficacy predict their interprofessional competencies?
- H_3_: Students’ perceived team efficacy positively predicts interprofessional competencies. Effective teams create environments for role clarification, communication, and mutual feedback, which are key components of interprofessional competencies [5].
- RQ4: Does students’ perceived team efficacy mediate the relationship between academic motivation and interprofessional competencies?
- H_4_: Students’ perceived team efficacy partially mediates the relationship between academic motivation and interprofessional competencies. We hypothesize partial rather than full mediation because motivated learners engage in both individual learning (e.g., independent study, observation, reflection) and team-based learning. Motivation influences interprofessional competencies directly through individual pathways. It also operates indirectly through students’ perceived team efficacy by fostering engagement in team processes that satisfy needs for autonomy, competence, and relatedness [24, 25].

## Methods

### Study Design and Procedures

This cross-sectional study collected data at the conclusion of two four-week IPE modules (Fracture and Emerging Respiratory Infection) implemented within the Interprofessional Education and Collaborative Practice (IPECP) programme at the University of Hong Kong, in collaboration with the Hong Kong Polytechnic University.

Both modules were designed following the four-part Preparation, Readiness Assurance, Application, and Enrichment (PRAE) model of the IPECP programme [26]. PRAE activities include active learning strategies, team-based collaborative case simulations, role clarification exercises, and team-based problem-solving activities, purposely aligned with the core competencies articulated by the IPEC [5]. Full details of these activities are described in Ganotice et al. [26]. The two modules shared identical pedagogical structure, duration, team composition, and outcome instrumentation, differing only in clinical content. Participants were therefore pooled across modules for the primary analyses.

Students were randomly assigned to interprofessional teams of 10 to 11 members, ensuring diverse representation from all participating disciplines, with at least one student from each discipline per team. At the end of their IPE module, participants were invited to complete an anonymous online survey via Qualtrics.

This study was conducted under Human Research Ethics Committee approval number EA240112. The umbrella protocol covers an experimental mixed-methods comparison of gamified and traditional IPE in two other IPECP modules; the present study uses the same student population and data-collection procedure in the Fracture and Emerging Respiratory Infection modules, without gamification or experimental manipulation. Participation was entirely voluntary and did not affect academic assessment. Informed consent was obtained from all participants prior to data collection. All procedures were in accordance with the 2024 Declaration of Helsinki.

### Measures

Academic knowledge motivation (AMK) and academic accomplishment motivation (AMA) were assessed using their respective two-item subscales from the Short Academic Motivation Scale (SAMS) [27]. Participants were asked “*Why do you go to college?”* and responded on a seven-point scale ranging from 1 (“does not correspond at all”) to 7 (“corresponds exactly”). AMK items: (1) “*For the pleasure I experience when I discover new things never seen before*”; (2) “*Because my studies allow me to continue to learn about many things that interest me*.” AMA items: (1) “*For the pleasure that I experience while I am surpassing myself in one of my personal accomplishments*”; (2) “*Because college allows me to experience a personal satisfaction in my quest for excellence in my studies.*” Higher mean scores reflect greater motivation for acquiring academic knowledge and for accomplishment, respectively. Given the two-item structure of each subscale, reliability was estimated using the Spearman-Brown coefficient (r_sb), which is the methodologically appropriate estimate for two-item scales [28] and is mathematically equivalent to Cronbach’s alpha in this case (AMK: r_sb = 0.92; AMA: r_sb = 0.92).

Perceived team efficacy (TE) was measured using the Team Efficacy Questionnaire [29]. This three-item questionnaire assesses participants’ perceptions of how effectively their team functioned during the IPE module. An example item is “*I am satisfied with the performance of my team*.” Items were rated on a five-point scale ranging from 1 (“strongly disagree”) to 5 (“strongly agree”). Higher mean scores indicate greater perceived team efficacy (α = 0.93).

Interprofessional competencies were evaluated using the IPEC Competency Self-Assessment Tool [23]. The scale comprises 42 items across four domains: Values and Ethics (VE), Roles and Responsibilities (RR), Interprofessional Communication (IC), and Teams and Teamwork (TT). Example items include “*Communicate my roles and responsibilities clearly to patients*,* families*,* and other professionals*” and “*Choose effective communication tools and techniques to facilitate discussions that enhance team function*.” Items were rated on a five-point scale ranging from 1 (“strongly disagree”) to 5 (“strongly agree”). Higher mean scores indicate greater perceived interprofessional competence (α = 0.97–0.98 across subdomains).

All measures were administered at the end of the two four-week IPE modules, after teams had completed all PRAE activities, ensuring sufficient team interaction for efficacy beliefs to emerge while capturing motivation during active engagement.

### Data Analysis

Prior to analysis, data were screened for univariate and multivariate outliers, missing values, and violations of assumptions. Variance inflation factors (VIF) were calculated for all predictors. Values ranged from VIF = 1.42 to VIF = 2.84, all falling within conventional thresholds for acceptable collinearity (VIF < 5; [30]), indicating that predictors were near-independent and that regression coefficients were estimated without meaningful variance inflation. Heteroscedasticity-consistent standard errors were employed to ensure robust inference. Descriptive and correlational (Pearson’s *r*) analyses were performed using the full sample (*n* = 144).

Mediation analyses were conducted using IBM SPSS version 26.0 with the PROCESS macro version 4.2 (Model 4) [31]. PROCESS Model 4 implements ordinary least squares multiple regression for each pathway of the mediation model: (*i*) regression of the mediator (perceived team efficacy) on the independent variable (AMK or AMA) and covariates, yielding the a path; (*ii*) regression of each outcome (VE, RR, IC, TT) on the mediator, the independent variable, and covariates simultaneously, yielding the b path and the direct effect c’; and (*iii*) computation of the indirect effect as the product a × b. We specified eight indirect pathways to test the mediating role of perceived team efficacy across both motivation subtypes and all four IPEC competency domains.

Bias-corrected bootstrap procedures with 5,000 resamples generated 95% confidence intervals for all indirect effects, while delta method confidence intervals were used for direct and total effects [32]. Mediation was considered statistically significant when bootstrap confidence intervals excluded zero. All models included age, gender, and discipline as demographic covariates to control for potential confounding effects. All significance tests used a two-tailed alpha level of 0.05 as the threshold for statistical significance.

## Results

Of the 279 enrolled interprofessional undergraduate students, 144 (response rate: 51.6%) consented and completed the post-module survey. Among them, 69 (47.9%) were female and 75 (52.1%) were male. Students were enrolled in Medicine (MBBS; *n* = 24; 16.7%) and Nursing (*n* = 90; 62.5%) programmes at the University of Hong Kong, and Physiotherapy (*n* = 30; 20.8%) at the Hong Kong Polytechnic University (Table 1).

**Table 1:** Sample characteristics (*n* = 144)

Variable	Category	*n*	%
Gender			
	Female	69	47.9
	Male	75	52.1
Discipline			
	Nursing	90	62.5
	Physiotherapy	30	20.8
	MBBS (Medicine)	24	16.7

The mean age of the participants is 21.57 (SD = 2.48), with the minimum age of 19 and a maximum of 30 years old; MBBS = Bachelor of Medicine, Bachelor of Surgery

### Preliminary Analyses

Correlation analyses revealed significant positive associations among academic motivation dimensions (AMK and AMA), team efficacy (TE), and all four IPEC competency domains: VE, RR, IC, and TT, ranging from *r* = 0.51 to *r* = 0.97 (all *p* < 0.001; Table 2). These preliminary correlational associations are consistent with the hypothesised relationships subsequently tested in the multiple-regression mediation model (Sect. Testing the Mediation Model).

**Table 2:** Descriptive statistics and correlations among team efficacy, academic motivation knowledge, academic motivation achievement, interprofessional education competence

Variable	TE	AMK	AMA	VE	RR	IC	TT
TE	-	0.52	0.53	0.51	0.52	0.51	0.51
AMK		-	0.87	0.70	0.71	0.71	0.71
AMA			-	0.67	0.67	0.66	0.65
VE				-	0.93	0.90	0.89
RR					-	0.95	0.97
IC						-	0.95
TT							-
Mean	4.14	5.31	5.27	3.97	3.96	3.97	3.96
*SD*	0.76	1.07	1.08	0.74	0.74	0.71	0.72
Kurtosis	4.55	0.26	1.06	0.90	-0.45	-0.33	-0.31
Skewness	-1.62	-0.52	-0.56	-0.58	-0.33	-0.31	-0.29
Cronbach’s alpha	0.93	0.92	0.92	0.98	0.97	0.97	0.98

*TE *team efficacy, *AMK *academic motivation knowledge, *AMA *academic motivation accomplishment, *VE *values and ethics, *RR *roles and responsibilities, *IC *interprofessional communication, *TT *teamwork and team-based practice. All correlations are significant at *p* < 0.001

### Testing the Mediation Model

#### RQ1: Direct Effects of Academic Motivation on Interprofessional Competencies (Path c’)

**Fig. 2:**
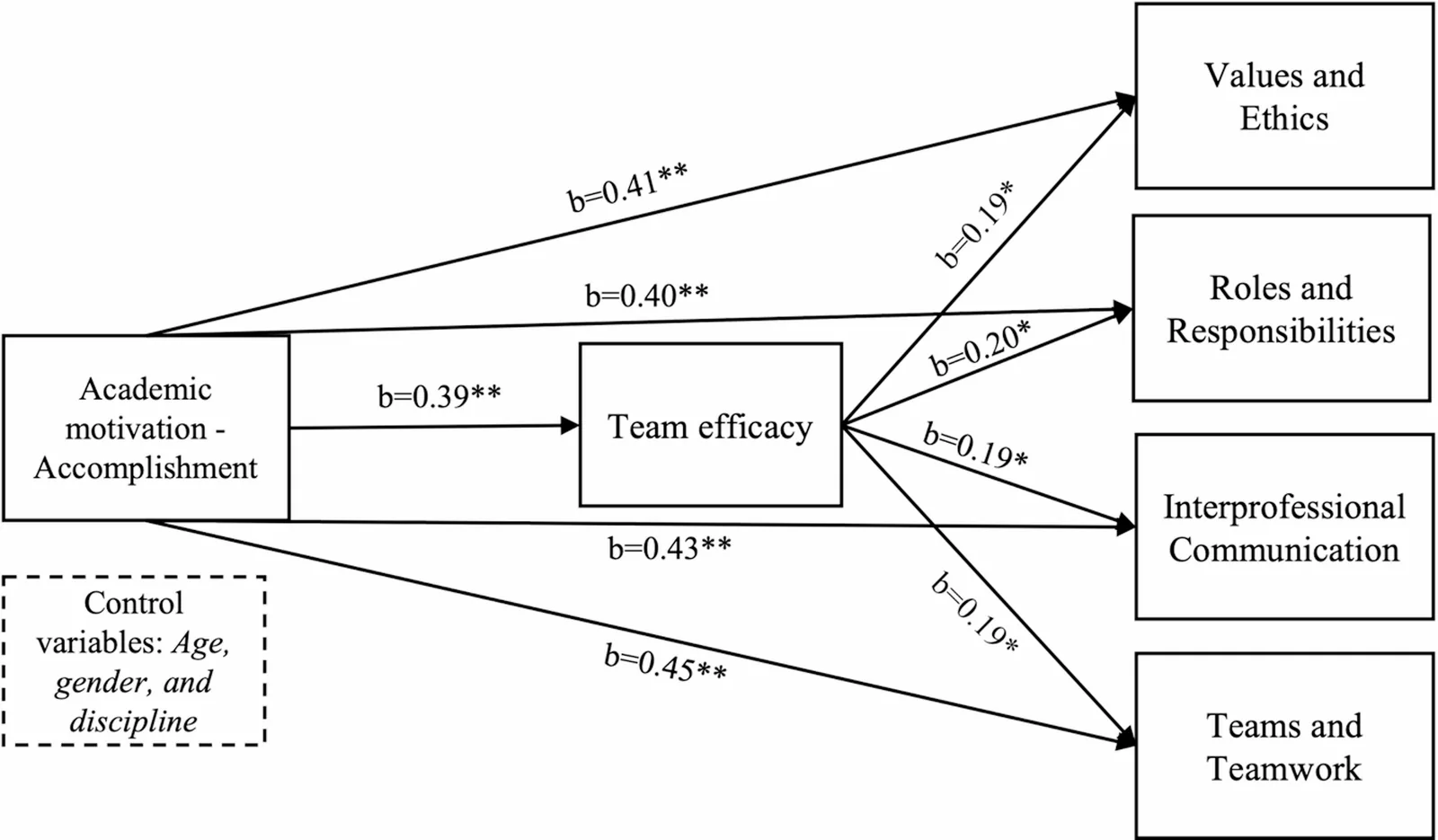
Mediation model with academic accomplishment motivation (AMA) as predictor, perceived team efficacy as mediator, and the four IPEC competency domains as outcomes.*Note*: Unstandardized path coefficients are shown. All paths are positive and significant. Indirect effects of AMA through perceived team efficacy: Values and ethics, b = 0.07 (95% CI [0.00, 0.18]); Roles and responsibilities, b = 0.07 (95% CI [0.02, 0.15]); Interprofessional communication, b = 0.07 (95% CI [0.01, 0.14]); Teams and teamwork, b = 0.07 (95% CI [0.01, 0.16]). All indirect effects significant. * *p* < 0.05. ** *p* < 0.001. Bootstrapped 95% CIs based on 5,000 samples (PROCESS Model 4, *N* = 144)

AMA (Academic Accomplishment Motivation) significantly predicted all four IPEC domains (see Table 3; Fig. 2): VE (Values and Ethics; *b* = 0.41, SE = 0.05, *p* < 0.001, 95% CI [0.31, 0.51]), RR (Roles and Responsibilities; *b* = 0.40, SE = 0.05, *p* < 0.001, 95% CI [0.30, 0.49]), IC (Interprofessional Communication; *b* = 0.43, SE = 0.04,*p* < 0.001, 95% CI [0.35, 0.52]), and TT (Teams and Teamwork; *b* = 0.45, SE = 0.04, *p* < 0.001, 95% CI [0.37, 0.54]).

**Table 3:** Mediation analysis results: direct, indirect, and total effects

		Outcome	Unstand. Est.	Std. Est.	SE	*p*	LLCI	ULCI
Direct Effects								
AMA	→	VE	0.410	0.599	0.048	< 0.001	0.314	0.506
AMA	→	RR	0.398	0.585	0.048	< 0.001	0.304	0.493
AMA	→	IC	0.433	0.664	0.042	< 0.001	0.350	0.515
AMA	→	TT	0.452	0.676	0.042	< 0.001	0.368	0.536
AMA	→	TE	0.359	0.509	0.051	< 0.001	0.258	0.460
AMK	→	VE	0.403	0.581	0.052	< 0.001	0.301	0.505
AMK	→	RR	0.405	0.587	0.050	< 0.001	0.305	0.504
AMK	→	IC	0.393	0.595	0.049	< 0.001	0.296	0.490
AMK	→	TT	0.418	0.617	0.049	< 0.001	0.321	0.515
AMK	→	TE	0.390	0.545	0.050	< 0.001	0.291	0.489
TE	→	VE (IV: AMA)	0.194	0.200	0.069	0.006	0.057	0.331
TE	→	RR (IV: AMA)	0.204	0.212	0.069	0.004	0.068	0.340
TE	→	IC (IV: AMA)	0.187	0.202	0.068	0.007	0.052	0.322
TE	→	TT (IV: AMA)	0.189	0.199	0.069	0.007	0.048	0.312
TE	→	VE (IV: AMK)	0.181	0.186	0.073	0.015	0.036	0.325
TE	→	RR (IV: AMK)	0.181	0.188	0.071	0.012	0.040	0.322
TE	→	IC (IV: AMK)	0.149	0.161	0.069	0.034	0.011	0.286
TE	→	TT (IV: AMK)	0.145	0.153	0.070	0.039	0.007	0.283
**Indirect Effects**								
AMA → TE	→	VE	0.070	0.102	0.047	---	0.001	0.179
AMA → TE	→	RR	0.073	0.108	0.033	---	0.018	0.147
AMA → TE	→	IC	0.067	0.103	0.033	---	0.013	0.143
AMA → TE	→	TT	0.068	0.101	0.037	---	0.012	0.156
AMK → TE	→	VE	0.070	0.102	0.050	---	-0.002	0.189
AMK → TE	→	RR	0.071	0.102	0.037	---	0.009	0.155
AMK → TE	→	IC	0.058	0.088	0.034	---	-0.004	0.133
AMK → TE	→	TT	0.057	0.084	0.039	---	-0.002	0.152
**Total Effects**								
AMA → TE	→	VE	0.480	0.701	0.043	< 0.001	0.396	0.564
AMA → TE	→	RR	0.472	0.693	0.042	< 0.001	0.388	0.555
AMA → TE	→	IC	0.433	0.664	0.042	< 0.001	0.350	0.515
AMA → TE	→	TT	0.452	0.676	0.042	< 0.001	0.368	0.536
AMK → TE	→	VE	0.474	0.683	0.044	< 0.001	0.301	0.505
AMK → TE	→	RR	0.475	0.689	0.043	< 0.001	0.391	0.560
AMK → TE	→	IC	0.451	0.683	0.041	< 0.001	0.369	0.533
AMK → TE	→	TT	0.474	0.700	0.042	< 0.001	0.392	0.556

*N* = 144. Unstand. Est. = unstandardized estimate; Std. Est. = standardized estimate; SE = standard error; LLCI = lower level confidence interval; ULCI = upper level confidence interval; IV = independent variable; AMA = academic accomplishment motivation; AMK = academic knowledge motivation; TE = team efficacy; VE = values/ethics; RR = roles/responsibilities; IC = interprofessional communication; TT = team and teamwork. Bootstrapped SE and 95% CIs for indirect effects were based on 5,000 bootstrap samples. Heteroscedasticity-consistent standard error and covariance matrix estimator were used

**Fig. 3:**
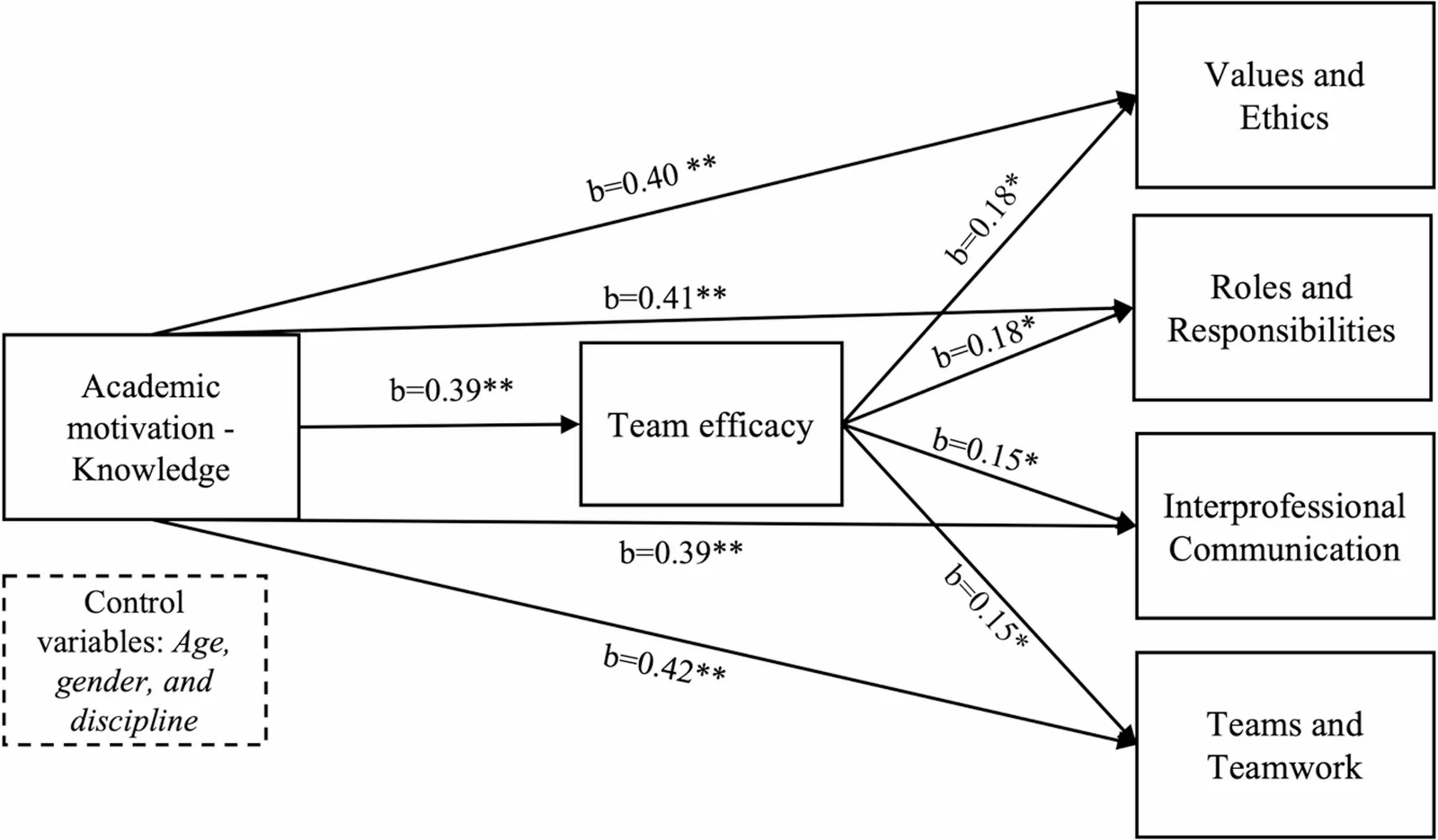
Mediation model with academic knowledge motivation (AMK) as predictor, perceived team efficacy as mediator, and the four IPEC competency domains as outcomes.*Note*: Unstandardized path coefficients shown. All direct paths are positive and significant. Indirect effects of AMK through perceived team efficacy: Values and ethics, b = 0.07 (95% CI [− 0.00, 0.19]); Roles and responsibilities, b = 0.07 (95% CI [0.01, 0.16])*; Interprofessional communication, b = 0.06 (95% CI [− 0.00, 0.13]); Teams and teamwork, b = 0.06 (95% CI [− 0.00, 0.15]). * *p* < 0.05. ** *p* < 0.001. Bootstrapped 95% CIs based on 5,000 samples (PROCESS Model 4, *N* = 144)

Similarly, AMK (Academic Knowledge Motivation) significantly predicted all four IPEC domains (see Table 3; Fig. 3): VE (*b* = 0.40, SE = 0.05, *p* < 0.001, 95% CI [0.30, 0.51]), RR (*b* = 0.41, SE = 0.05, *p* < 0.001, 95% CI [0.31, 0.50]), IC (*b* = 0.39, SE = 0.05, *p* < 0.001, 95% CI [0.30, 0.49]), and TT (*b* = 0.42, SE = 0.05 *p* < 0.001, 95% CI [0.32, 0.52]).

These findings support H_1_, indicating that higher levels of academic motivation are directly associated with greater interprofessional competence, even when accounting for age, gender, and discipline (see Supplementary Table 1).

#### RQ2: Effects of Academic Motivation on Team Efficacy (Path a)

Both dimensions of academic motivation significantly predicted team efficacy, supporting H_2_. AMK (Academic Knowledge Motivation) demonstrated a significant positive effect on team efficacy (*b* = 0.39, SE = 0.05, *p* < 0.001, 95% CI [0.29, 0.489]), as did AMA (Academic Accomplishment Motivation; *b* = 0.36, SE = 0.05, *p* < 0.001, 95% CI [0.26, 0.46]). AMK showed a slightly stronger effect (*b* = 0.39) compared to AMA (β = 0.36), suggesting that knowledge-oriented students may engage more actively in team-building behaviors. These findings suggest that learners with higher motivation—whether driven by knowledge acquisition or achievement goals—tend to perceive their teams as more effective and capable.

#### RQ3: Effects of Students’ Perceived Team Efficacy on Interprofessional Competencies (Path b)

Student’s perceived team efficacy significantly predicted all four IPEC competency domains, fully supporting H_3_. Across the mediation models, higher perceived team efficacy was consistently associated with greater competence in VE (Values and Ethics; *b* = 0.18–0.19, *p* < 0.05), RR (Roles and Responsibilities; *b* = 0.18–0.20, *p* < 0.05), IC (Interprofessional Communication; *b* = 0.15–0.19, *p* < 0.05), and TT (Teams and Teamwork; *b* = 0.15–0.19, *p* < 0.05). These results demonstrate that perceived team efficacy could enhance learners’ interprofessional skills and collaborative abilities across all competency domains.

#### RQ4: Indirect Effects Through Team Efficacy (Mediation Analysis)

The mediation analysis revealed differential patterns for the two motivation dimensions, providing partial support for H_4_.

The indirect effect of AMK (Academic Knowledge Motivation) on interprofessional competencies through team efficacy was significant only for RR (Roles and Responsibilities; *b* = 0.07, SE = 0.04, 95% CI [0.01,0.16]). The bootstrap confidence intervals for the other three domains included zero, indicating non-significant indirect effects: VE (Values and Ethics; 95% CI [-0.00,0.19]), IC (Interprofessional Communication; 95% CI [-0.00,0.13]), and TT (Teams and Teamwork; 95% CI [-0.00,0.15]). This pattern suggests that knowledge-motivated students develop role clarity competencies partially through enhanced team efficacy, while their development of ethical values, communication skills, and teamwork abilities occurs primarily through direct individual learning pathways rather than team-mediated processes.

In contrast, AMA demonstrated significant indirect effects through team efficacy across all four IPEC domains: VE (*b* = 0.07, SE = 0.05, 95% CI [0.00,0.18]), RR (*b* = 0.07, SE = 0.03, 95% CI [0.02,0.15]), IC (*b* = 0.07, SE = 0.03, 95% CI [0.01,0.14]), and TT (*b* = 0.07, SE = 0.04, 95% CI [0.01,0.16]). All confidence intervals excluded zero, confirming significant mediation.

## Discussion

### The Motivational Foundation of Collaborative Learning

Academic motivation, including both knowledge motivation and achievement motivation, was positively associated with all four IPE competency subdomains (values and ethics, roles and responsibilities, interprofessional communication, and teams and teamwork). This aligns with prior research indicating that motivated learners are more likely to engage actively in learning activities that foster skill development [9, 33]. Such engagement is crucial for the acquisition of interprofessional competencies necessary for effective healthcare practice.

In addition, academic motivation was also positively associated with perceived team efficacy. This supports earlier findings that motivation influences perceptions of team effectiveness [12, 34]. Motivated learners tend to perceive their teams as more capable, which in turn may enhance team cohesion and individual responsibility, both of which are essential elements for successful collaboration [35]. These perceptions are particularly relevant in interprofessional settings where trust and shared efficacy are vital for teamwork [36].

### Team Efficacy as a Collective Bridge Mechanism

Beyond its direct associations with academic motivation, perceived team efficacy showed positive associations with all four IPEC competency domains. In line with the broader team science literature, perceptions of team effectiveness are linked to role clarity, open communication, and sustained cooperative behavior over time [34, 37]. We posit that students who perceive higher team efficacy may be more likely to engage in specific competency-building behaviors. For example, members clarify professional roles across disciplines (roles and responsibilities), engage in difficult conversations about values conflicts (values and ethics), surface knowledge gaps through questioning (interprofessional communication), and provide constructive peer feedback (teams and teamwork). This supportive climate reflects a reduction in the interpersonal risks typically associated with cross-disciplinary learning [13, 38].

The mediation analyses revealed a theoretically meaningful asymmetry between the two motivation subtypes, partially supporting H4. Students’ perceived team efficacy mediated the association between AMA and all four IPEC domains, whereas for AMK, mediation was significant only for roles and responsibilities. This pattern is consistent with the theoretical framing developed in Sect. Theoretical Framework. Accomplishment motivation, grounded in the competence and relatedness needs articulated by SDT [7], is associated with greater orientation toward team performance, suggesting that perceived team efficacy may serve as a channel linking AMA to competency development across all four domains. Knowledge motivation, more anchored in individual information-seeking and intellectual curiosity, appears linked to competency development primarily through direct learning pathways rather than team-mediated processes [6]. The exception for roles and responsibilities is interpretable: this domain centers on factual understanding of other professions’ scopes, training, and decision-making authority, making it more amenable to knowledge acquisition through team interaction than the more behaviorally enacted competencies of communication, values negotiation, and teamwork [5]. Given the high AMK-AMA intercorrelation (*r* = 0.87) and the proximity of several AMK indirect-effect confidence intervals to zero, this asymmetric pattern warrants replication in larger samples with longitudinal designs.

### Implications for Theory and Educational Practice

Overall, these findings underscore the importance of fostering both motivation and perceptions of team efficacy within interprofessional education contexts. While high motivation can stimulate engagement and positive perceptions of team effectiveness, strengthening team efficacy was directly associated with the development of core interprofessional competencies.

These findings extend self-determination theory beyond individual learning contexts by demonstrating how personal motivation generates collective team states. The mediation through team efficacy suggests that motivation’s influence on learning may be partly social. That is, it may operate through the interpersonal dynamics and collaborative behaviors that motivated individuals create within team settings. This represents an important theoretical advancement, showing that SDT’s emphasis on relatedness needs may manifest not just in individual learning contexts, but in how motivated learners contribute to team functioning.

These findings suggest several practical directions for IPE curriculum design. Accomplishment-motivated students may benefit from structured team-level goal-setting activities, where shared, tangible goals channel achievement drive into collaborative team processes [39]. Knowledge-motivated students may be better served by inquiry-based tasks that require cross-disciplinary information exchange, particularly for developing roles-and-responsibilities competencies. Across both subtypes, facilitation approaches that offer student choice and acknowledge diverse learning goals may help sustain autonomous engagement throughout IPE modules. These recommendations are offered pending replication in larger and more diverse samples.

### Limitations and Future Directions

Several limitations warrant consideration. First, the cross-sectional design precludes causal inference about the proposed mediation pathways and constrains interpretation of temporal precedence; longitudinal designs are needed to clarify the directional dynamics among motivation, perceived team efficacy, and competency development. Second, the present sample’s Cronbach’s alphas for the IPEC subdomains (α ≥ 0.97) and the inter-subdomain correlations (*r* = 0.89–0.97) exceed thresholds at which Tavakol and Dennick [40] recommend interpretive caution. Similarly, the AMK-AMA intercorrelation (*r* = 0.87) indicates substantial shared variance between the two motivation subtypes, and the differential mediation pattern observed across subtypes should therefore be read as suggestive rather than as evidence of fully separable mechanisms. While we used the IPEC tool in its validated form, these values likely reflect a mixture of construct homogeneity, item overlap within the published scale, and common-method variance from single-occasion self-report. Findings across the four IPEC subdomains should be read as closely related aspects of a broader competency construct rather than fully independent outcomes. Third, perceived team efficacy was operationalised at the individual rather than the collective level; results should be interpreted as individual perceptions of team functioning rather than as a measured collective efficacy belief. Team-level aggregation was not feasible given the within-team sample sizes available, and future multilevel designs are needed to test the construct as a shared team state. Fourth, the sample consisted of students from Hong Kong, which limits the generalisability of the findings across cultural and regional contexts.

Future research should employ longitudinal designs and larger samples to strengthen causal inference and generalisability. Examining abbreviated versions of the IPEC tool, or scoring approaches that combine the four subdomains, would clarify whether the high inter-construct correlations observed here are a feature of this sample or of the instrument itself. Investigating additional mediators and moderators, such as self-efficacy, prior IPE experience (e.g., Wong et al., [41]) or institutional support, would also provide a more nuanced understanding of competency development. Comparative studies across diverse educational settings will be important for validating these patterns and informing culturally responsive IPE design.

## Conclusion

This study tested whether perceived team efficacy mediates the relationship between two subtypes of academic motivation and four interprofessional competency domains. Across all four domains, accomplishment motivation was associated with both team-mediated and individual pathways; knowledge motivation was associated primarily with direct learning, with perceived team efficacy mediating only the roles and responsibilities domain. The indirect effects through perceived team efficacy were modest in size (*b* ≈ 0.06–0.07), as were the direct associations between perceived team efficacy and IPEC (*b* = 0.15–0.20). The two motivation subtypes were also highly correlated in this sample (*r* = 0.87), and the differential mediation pattern should therefore be read as one of degree rather than kind.

Within these constraints, the findings suggest that IPE curricula may benefit from structuring team tasks to leverage the team-oriented expression of accomplishment motivation, while providing individual learning opportunities such as independent reading, observation, and structured reflection that engage knowledge-motivated students. Deliberate attention to building perceived team efficacy across both pathways remains warranted, given its consistent direct associations with competency development across all four IPEC domains.

## Supplementary Information

Below is the link to the electronic supplementary material.

Supplementary Material 1

## Data Availability

The data that support the findings of this study can be requested from the corresponding author, FAG, upon reasonable request.
