# Supplementary material for: Do Students’ Motivation Influence Interprofessional Competencies? The Mediating Role of Team Efficacy
Source: Med Sci Educ. 2026 Jun 23;36(4):1759–71. doi: 10.1007/s40670-026-02789-2 (PMC13582714; doi:10.1007/s40670-026-02789-2)
Supplement: Supplementary file 1 [file 40670_2026_2789_MOESM1_ESM.docx]

**Supplementary Table 1**

*Regression results for covariates predicting the outcome variables*

|  |  | **Outcome** | **Unstand. Est.** | **Std. Est.** | **SE** | ***p*** | **LLCI** | **ULCI** |
| --- | --- | --- | --- | --- | --- | --- | --- | --- |
| **Covariates (IV: AMK)** |  |  |  |  |  |  |  |  |
| Age | → | VE | -0.020 | -0.067 | 0.020 | .320 | -0.059 | 0.019 |
| Gender | → | VE | 0.085 | 0.057 | 0.091 | .353 | -0.095 | 0.264 |
| Discipline | → | VE | -0.075 | -0.083 | 0.622 | .232 | -0.198 | 0.048 |
| Age | → | RR | -0.015 | -0.051 | 0.019 | .436 | -0.053 | 0.023 |
| Gender | → | RR | 0.111 | 0.076 | 0.089 | .209 | -0.063 | 0.287 |
| Discipline | → | RR | -0.053 | -0.059 | 0.061 | .386 | -0.173 | 0.067 |
| Age | → | IC | -0.008 | -0.028 | 0.019 | .677 | -0.045 | 0.029 |
| Gender | → | IC | 0.081 | 0.058 | 0.086 | .351 | -0.090 | 0.251 |
| Discipline | → | IC | -0.009 | -0.011 | 0.059 | .878 | -0.126 | 0.108 |
| Age | → | TT | -0.021 | -0.072 | 0.019 | .273 | -0.058 | 0.017 |
| Gender | → | TT | 0.097 | 0.067 | 0.086 | .264 | -0.074 | 0.268 |
| Discipline | → | TT | -0.041 | -0.047 | 0.059 | .489 | -0.158 | 0.076 |
|  |  |  |  |  |  |  |  |  |
| **Covariates (IV: AMA)** |  |  |  |  |  |  |  |  |
| Age | → | VE | -0.014 | -0.047 | 0.019 | .472 | -0.052 | 0.024 |
| Gender | → | VE | 0.045 | 0.030 | 0.089 | .617 | -0.131 | 0.220 |
| Discipline | → | VE | -0.066 | -0.073 | 0.061 | .276 | -0.186 | 0.053 |
| Age | → | RR | -0.009 | -0.031 | 0.019 | .631 | -0.047 | 0.029 |
| Gender | → | RR | 0.075 | 0.051 | 0.088 | .394 | -0.099 | 0.250 |
| Discipline | → | RR | -0.044 | -0.049 | 0.060 | .464 | -0.163 | 0.075 |
| Age | → | IC | 0.024 | 0.079 | 0.024 | .307 | -0.022 | 0.071 |
| Gender | → | IC | 0.014 | 0.009 | 0.109 | .901 | -0.203 | 0.230 |
| Discipline | → | IC | 0.000 | 0.000 | 0.060 | .913 | -0.118 | 0.118 |
| Age | → | TT | -0.015 | -0.050 | 0.019 | .449 | -0.053 | 0.023 |
| Gender | → | TT | 0.067 | 0.089 | 0.890 | .454 | -0.109 | 0.243 |
| Discipline | → | TT | -0.031 | -0.036 | 0.060 | .604 | -0.151 | 0.088 |
